# Rare presentation of annular and polypoid heterotopic gastric mucosa in duodenum

**DOI:** 10.1002/jgh3.12434

**Published:** 2020-12-31

**Authors:** Takayoshi Kiba, Naoki Kotoh, Masahiro Tsuboi

**Affiliations:** ^1^ Department of Internal Medicine Saiseikai Kibi Hospital Okayama Japan; ^2^ Department of Life Sciences, Faculty of Science Okayama University of Science Okayama Japan; ^3^ Department of Neurosugery Saiseikai Kibi Hospital Okayama Japan

**Keywords:** duodenum, heterotopic gastric mucosa, rare presentation

## Abstract

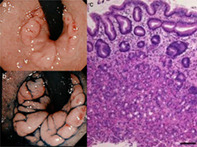

In this case, Esophagogastroduodenoscopy showed an approximately 25‐mm diameter annular and polypoid mucosa at the opposite side of Pylorus ring in first part of duodenum. The biopsy specimens were diagnosed with the heterotopic gastric mucosa (HGM) pathologically. This is a rare presentation of annular and polypoid HGM in duodenum. This may indicate the needs for other documents as well as analysis regarding the mechanism for the development of HGM in duodenum.

A 69‐year‐old man presented with epigastralgia of a duration of 1 month. His past medical history was notable for hypertension. The patient underwent routine nasal esophagogastroduodenoscopy (EGD) (GIF‐H190N, Olympus Optical Co, Japan). EGD showed an annular and polypoid mucosa measuring approximately 25 mm in diameter at the opposite side of the Pylorus ring in the first part of the duodenum (Fig. 1a,b). His serologic test for *Helicobacter pylori* antibody was negative. There was no evidence of hemorrhage in the stomach and duodenum. Histology showed gastric heterotopic tissues, containing fundic glands, with inflammatory cells. The biopsy specimens were diagnosed as heterotopic gastric mucosa (HGM) pathologically (Fig. 1c). These were negative for dysplasia and malignancy.

**Figure 1:**
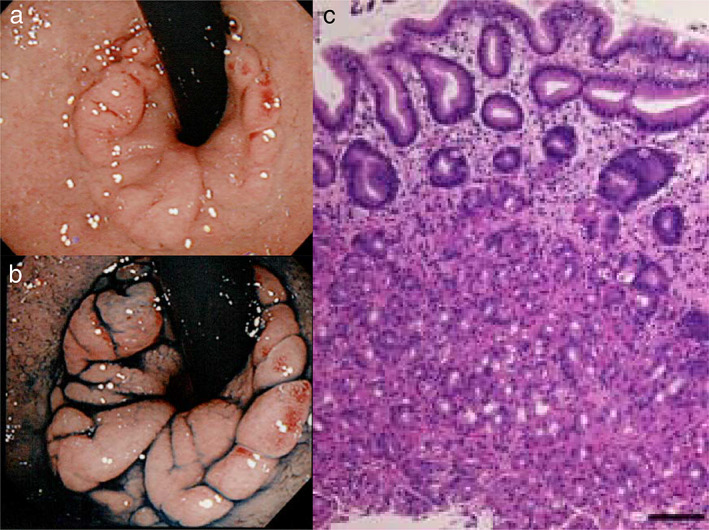
(a) An annular and polypoid HGM was observed in the duodenum detected by retroflex esophagogastroduodenoscopy. (b) Linked collar imaging with indigo carmine dye. (c) Hematoxylin and eosin stain of the biopsy specimens showed gastric heterotopic tissues, containing fundic glands, with inflammatory cells. (bar = 100 μm).

A previous study observed duodenal HGM in 8.9% of 28 210 patients who underwent EGD, and HGM was recognized as having solitary or multiple small nodules.^1^ HGM has often been reported to be asymptomatic. However, once the lesion has been detected, appropriate treatment, which will reduce the risk of complications, can be implemented.^2^ Clinical presentations of HGM, such as intestinal obstruction, bleeding, perforation, and fistula formation to adjacent structures, vary and depend on the location and size.^3^ It has also been reported that solitary large HGMs may be difficult to differentiate from malignant neoplasms such as carcinomas^4^; however, a differential diagnosis for it has yet to be established. Excessive surgery should be avoided because HGM is essentially a benign entity.^4^ However, if the HGM is large, it may still require laparoscopic or endoscopic resection. In this case, although the patient also received a second opinion, it was decided that endoscopic follow‐up was needed for the lesion.

In conclusion, this is a rare presentation of annular and polypoid HGM in the duodenum. This may indicate the need for other studies, as well as analysis, regarding the mechanism for the development of HGM in the duodenum.
